# Impaction of the Fourth Fold of the Large Colon of a Horse, Complicated by Strangulation of the Ileum in the Foramen of Winslow—Fatal

**Published:** 1902-07

**Authors:** John J. Repp

**Affiliations:** Veterinarian to the Experiment Station, Iowa State College, Ames, Ia.


### IMPACTION OF THE FOURTH FOLD OF THE LARGE COLON IN A HORSE, COMPLICATED BY STRANGULATION OF THE ILEUM IN THE FORAMEN OF WINSLOW—FATAL.

By John J. Repp, V.M.D.,
VETERINARIAN TO THE EXPERIMENT STATION, IOWA STATE COLLEGE, AMES, IA.

*Subject.* Bay gelding, used for driving, aged thirteen years, fifteen hands high; 1000 pounds weight.

*History.* Animal was seen by owner at 11 A.M., June 29, 1902, and appeared well. At 1 P.M., when attendant went to feed him the horse was found to be ill with colic. By way of treatment the owner had smoked his head and, as I afterward found, put an onion into his rectum. Owner said the horse had two good-sized passages of normal feces after his sickness was noticed. Was called at 3 P.M., and arrived half-hour later.

*Observations and Treatment.* Found the horse lying upon his side and pawing. He rose without urging. Peristalsis weak in small intestines; normal on right side; pulse, respiration, and mucous membranes normal; rectum empty; folding colon could not be felt; animal was in pain and pawed violently, almost continuously, while standing. There was no tympany.

A diagnosis of colic, probably due to exacerbation of chronic simple gastro-enteritis, was made. The pawing was very suggestive of impaction, but if the history was to be credited the attack was too acute for that. Five grains of morphine sulphate was given hypodermically. Then about three gallons of cold water was injected as high into the colon as possible. Part of this was expelled at once and part retained. No feces were passed. This was followed by a draught of an ounce of tincture of opium, 4 drachms of fluid extract of cannabis indica and 4 ounces of water. I left the animal at 4 P.M. in about the same condition that I found him. I had the owner procure three pints of raw linseed oil, with directions to give one-half of it as soon as he obtained it, which he did at 4.30 P.M. I also prescribed 1 ounce of fluid extract of cannabis indica and 4 drachms of Pearson's creolin, to be given in a little water, as a drench, at 5 P.M. if the horse still showed no marked improvement. This dose was given. The owner reported to me at 6 P.M. that the horse was no better. I arrived at the stable at 7 P.M. and found the horse quite violent. He pawed when standing, and struggled, rolled, and groaned when lying. The pulse and respiration were accelerated, the nostrils dilated, the mucous membranes injected; no feces and but little flatus had been passed since my call. Peristalsis was very weak on the right side and absent on the left. There was considerable tympany upon the left side, but it was not alarming in its manifestations. Contrary to my belief that the puncture really ought to be made upon the left side, I introduced a trocar into the right flank, but failed to get any gas, either because there was no gas in the cæcum or because I failed to penetrate it. Realizing from the beginning of my second visit that the case was a desperate one, I now told the owner that I would have to use radical measures if there was to be any hope of putting death off beyond an hour or two. With his consent I gave 15 grains of barium chloride in solution in 2 drachms of water into the jugular vein, and followed it immediately by 1.5 grains of strychnine sulphate subcutaneously, to guard against the depressing effect of the barium chloride. The horse almost immediately became more active than ever; peristalsis became active for the first time, and at a considerable distance loud borborygmi could be heard; large quantities of flatus were expelled with considerable force; the strychnine early asserted itself, and there was well-developed strychnine tetanus; the pulse was about 80 and strong; respirations 30 and forceful; the horse walked about in a circle when on his feet, and frequently lay down and rose again. After fifty minutes had passed without the appearance of any feces, I gave 10 grains more of barium chloride into the jugular. Peristalsis very soon became more prominent, and the expulsion of flatus was rendered more active. After an hour of waiting after the second dose without the passage of feces, only a few small masses of mucopurulent matter having been expelled, 5 grains more of barium chloride was given into the jugular. This manifested its action in a very short time by increased peristalsis and renewed expulsion of gas. But still no feces. It was now 11 o'clock, and the barium chloride having failed to act and the horse showing a tendency to become quiet, I gave 1 pint of raw linseed oil, and, as the heart appeared rather weak, gave hypodermically 1 drachm of fluid extract of digitalis. I left the animal quiet and with a fair share of strength, but gave an unfavorable prognosis, although I still had a hope that the oil might bring about a passage from the bowels. At 6 o'clock the next morning the owner telephoned me that the horse was apparently the same as when I left him the evening before. I went to see him, found he had had no passage of feces, and that his heart was quite rapid and very weak. I prescribed strychnine sulphate 3 grains, cocaine hydrochlorate 10 grains, water 3 ounces, to be given a half-ounce every two hours, with the hope of keeping up strength until the oil might move the bowels. At noon the owner telephoned me that the horse was lying quietly and had been all forenoon, but that no feces had been passed. An hour later he informed me that the horse had just died.

*Autopsy, made four hours after death.* The cæcum and the large colon were slightly displaced, but this probably occurred post-mortem, as the carcass was conveyed two miles. The floating colon was empty. The folding colon was replete with alimentary matter throughout. Especially so was the fourth part, which was quite distended with matter, which was harder and dryer the more it approached the termination of the fold, at which point its passage was stopped. The mucous membrane of both colons at this point was dry, covered with a tenacious, dry, dark-colored secretion, and apparently inactive. So here was an impaction at the junction of the folding with the floating colon. The stomach was well filled with food, but the small intestine contained only a small amount of fluid matter, which was in the ileum. The ileum at a point eight inches anterior to the ileocæcal valve, and again at a point four feet anterior to that valve, was engaged in the foramen of Winslow, the intervening three feet four inches of ileum having passed through the foramen, and lay as a loop beyond. The strangulation was effectual. It required considerable manipulation and traction to withdraw the intestine from the foramen. The intestinal wall throughout the strangulated portion was greatly infiltrated with blood, and was the seat of a violent inflammatory reaction. There was a flaky croupous exudate upon the mucosa.

I believe that the impaction in the colon was the primary cause of the colic, and that if it had been uncomplicated it would have run a much longer and less violent course. The impacted food had not yet become nearly so hard and dry nor so tightly wedged in as in two other cases of impaction at this point with which I have had experience. Unfortunately, however, the passage of the ileum through the foramen of Winslow and its strangulation there rendered the attack acute. There was thus superimposed the clinical picture of strangulation of the intestine and a condition which failed to yield to treatment, and brought on a rapidly fatal result.

